# A Weighted Spatial-Spectral Kernel RX Algorithm and Efficient Implementation on GPUs

**DOI:** 10.3390/s17030441

**Published:** 2017-02-23

**Authors:** Chunhui Zhao, Jiawei Li, Meiling Meng, Xifeng Yao

**Affiliations:** College of Information and Communication Engineering, Harbin Engineering University, Harbin 150001, China; hgcljw@gmail.com (J.L.); meilingmeng@hrbeu.edu.cn (M.M.); xf.yao1020@gmail.com (X.Y.)

**Keywords:** anomaly detection, graphics processing units (GPUs), hyperspectral imaging, kernel mapping, spatial-spectral information, parallel processing

## Abstract

The kernel RX (KRX) detector proposed by Kwon and Nasrabadi exploits a kernel function to obtain a better detection performance. However, it still has two limits that can be improved. On the one hand, reasonable integration of spatial-spectral information can be used to further improve its detection accuracy. On the other hand, parallel computing can be used to reduce the processing time in available KRX detectors. Accordingly, this paper presents a novel weighted spatial-spectral kernel RX (WSSKRX) detector and its parallel implementation on graphics processing units (GPUs). The WSSKRX utilizes the spatial neighborhood resources to reconstruct the testing pixels by introducing a spectral factor and a spatial window, thereby effectively reducing the interference of background noise. Then, the kernel function is redesigned as a mapping trick in a KRX detector to implement the anomaly detection. In addition, a powerful architecture based on the GPU technique is designed to accelerate WSSKRX. To substantiate the performance of the proposed algorithm, both synthetic and real data are conducted for experiments.

## 1. Introduction

Hyperspectral imagery (HSI) is served as a three-dimensional cube which contains of two spatial dimensions and one spectral dimension. It provides the ability to distinguish the differences of ground-object spectra, so it has a wide range of applications in target detection [1]. Based on the availability of the prior information, the target detection algorithms can be divided into unsupervised and supervised ones. As accurate prior knowledge is difficult to obtain, unsupervised algorithms (anomaly detection algorithms) have drawn wide interest. Anomaly detection uses the differences between targets and the backgrounds to detect anomalies [2,3,4,5,6,7].

A widely used anomaly detection algorithm is the RX detector proposed by Reed and Yu [8]. The RX detector is a constant false alarm rate anomaly detection operator based on the assumption that hyperspectral image conforms to the multivariate Gaussian distribution. Actually, it determines a Mahalanobis distance between the pixel under test (PUT) and the background. Since the RX detector only makes use of the low-order statistics of hyperspectral data, it generally outputs an undesirable detection result when the distribution of ground materials is complex. To address this issue, the kernel RX (KRX) algorithm, a nonlinear version of the RX detector, was proposed by Kwon and Nasrabadi [9]. The KRX algorithm can utilize the nonlinear statistical information among hyperspectral bands effectively by mapping the spectral signal of original space into the high-dimensional feature space. In this way, it possesses more desirable detection accuracy compared to the RX algorithm. Unfortunately, the KRX detector is computationally very expensive due to the abundant non-linear kernel functions and inverse covariance matrices. This will affect the detection performance when the background kernel matrix degrades if background data are contaminated by anomalous pixels. Fortunately, reasonable use of spatial information and parallelism can solve the above problems, respectively.

With the increasing spatial resolution, spatial information has played a positive role in the field of hyperspectral images processing. It is critical to use spatial and spectral information for the effective increase of the detection performance in hyperspectral data [10]. Therefore, spatial information has been used for many algorithms, such as LSAD [11], DMSR [12], MD-L [13]. Generally, the method of combined spatial and spectral information has high computational complexity.

Many fast computational methods are used to accelerate hyperspectral image processing because of the high dimensionality of HSI [14]. Recently, with the continuous development of the Graphics Processing Units (GPUs), the powerful general-purpose computing power is revealed, which brings a new idea for the parallel optimization of anomaly detection. The attention of parallel processing for HSI on GPUs is increased [15,16,17,18]. The applications on GPUs is studied in accelerating target detection algorithm of Orthogonal Subspace Projection (OSP), and the hyperspectral remote sensing data is divided into blocks, which obtains a better performance [15]. GPUs implementation of hyperspectral anomaly detection based on a multivariate normal mixture model is proposed [17] and the experiment proves that the algorithm can be improved in terms of computational effectiveness.

In this paper, we develop a weighted spatial-spectral kernel RX (WSSKRX) algorithm and its efficient parallel version on GPUs. The proposed algorithm reconstructs the central pixel using the spatial neighborhood information and effectively reduces the interference of the abnormal pixel mixed in the background information. Then, WSSKRX achieves the spatial-spectral information integration by combining the original pixel with reconstructed pixel effectively. In addition, the parallel architecture is designed according to the characteristics of WSSKRX. This architecture is efficiently implemented on GPUs via the Compute Unified Device Architecture (CUDA) from NVIDIA. Experimental results show that the WSSKRX algorithm has better detection accuracy, and a much higher speedup is achieved compared to the Central Processing Unit (CPU) version of WSSKRX detector.

## 2. Methods

### 2.1. Anomaly Detection Methods

RX detector is the classical anomaly detection algorithm in hyperspectral imaging. Generally, there are two typical RX detector variants [19]: the global RX (GRX) detector and local RX (LRX) detector. The KRX detector obtains a better detection performance by exploiting the abundant nonlinear information among hyperspectral bands. In what follows, we review the RX and KRX algorithms.

#### 2.1.1. RX Detector

The RX detector, developed by Reed and Yu, is widely used in anomaly detection. Assume that **x***_i_* is the data sample vector and denoted by **x***_i_* = (*x_i_*_1_,*x_i_*_2_,…,*x_iL_*)^T^ where *L* is the total number of spectral bands. The two hypotheses model is given by:

(1) $$\{\begin{array}{l} H_{0}:\;x=b\;(\mathrm{Target}\;\mathrm{absent})\\ H_{1}:\;x=as+b\;(\mathrm{Target}\;\mathrm{present}) \end{array}$$

where *a* = 0 under **H**_0_ and *a* > 0 under **H**_1_. **b** is the background clutter, and **s** denotes the spectral signature of the target. The model assumes that the data arise from two normal probability density functions with the same covariance matrix but different means. In the **H**_0_ case, the background data are modeled as *N*(**μ**,**K**), and in the **H**_1_ case, the data are modeled as *N*(**μ** + **s**, **K**). The RX detector is defined by the following expression:

(2) $$\delta^{\mathrm{RXD}}(x_{n})={\left(x_{n}-\mu \right)}^{T}K^{-1}(x_{n}-\mu )$$

where **μ** is the mean of the background clutter data and **K** is the background covariance matrix. **K** and **μ** are defined by:

(3) $$K=\frac{1}{N}\sum_{i=1}^{N}(x(i)-\mu ){\left(x(i)-\mu \right)}^{T}$$

(4) $$\mu =\frac{1}{N}\sum_{i=1}^{N}x(i)$$

#### 2.1.2. Kernel RX Detector

**Figure 1:**
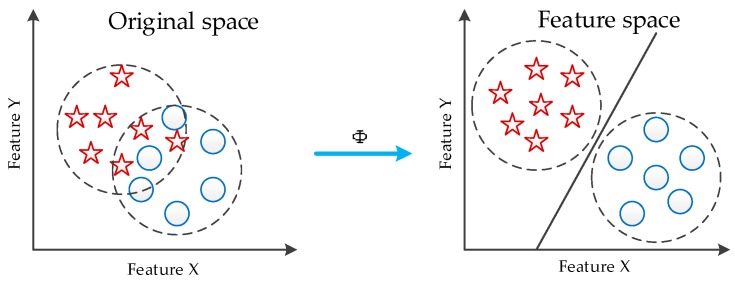
Illustration of nonlinear mapping.

The kernel RX (KRX) detector is a nonlinear version of the RX anomaly detection. As shown in Figure 1, the algorithm non-linearly maps the inputting signal to the high-dimensional feature space via kernel function, so the linear inseparable parts in original space can be separated linearly. Therefore, KRX algorithm has a better separation performance between background and target.

The original hyperspectral data **X***_b_* = [x_1_,x_2_,…,x*_M_*] is mapped to the high-dimensional feature space by a non-linear function Φ, Φ**X***_b_* = [Φ(x_1_),Φ(x_2_),…,Φ(x*_M_*)] is obtained. Then the corresponding KRX algorithm in the feature space is specified by:

(5) $$\mathrm{KRX}(\Phi (r))\;=\;(\Phi (r)-{\hat{\mu }}_{b\Phi }{)}^{T}{\hat{K}}_{b\Phi }^{-1}(\Phi (r)-{\hat{\mu }}_{b\Phi })$$

where ${\hat{K}}_{b\Phi }$ and ${\hat{\mu }}_{b\Phi }$ are the covariance matrix and mean of the background samples in the feature space, respectively. The centered inputting matrix in the feature space is expressed as $X_{\Phi c}$ = $[\Phi_{c}\left(x_{1}\right)\Phi_{c}\left(x_{2}\right),\ldots ,\Phi_{c}\left(x_{M}\right)],$ where $\Phi_{c}\left(x_{i}\right)=\Phi \left(x_{i}\right)\;{\hat{\mu }}_{b\Phi }$. Therefore, the covariance matrix can be represented by ${\hat{K}}_{b\Phi }=\frac{1}{M}X_{\Phi c}X_{\Phi c}^{T}$. Define the centered kernel matrix as $K_{c}=X_{\Phi c}^{T}X_{\Phi c}$. ${\hat{K}}_{b\Phi }$ and $K_{c}$ are real symmetric matrices, and they can be represented by its spectral decomposition as given by:

(6) $${\hat{K}}_{b\Phi }=V_{\Phi }\Lambda_{\Phi }V_{\Phi }^{T}$$

(7) $$K_{c}=Α\Lambda_{c}{Α}^{T}$$

where $V_{\Phi }$ and **A** are matrices whose columns are the eigenvectors, $\Lambda_{\Phi }$ and $\Lambda_{c}$ are diagonal matrices of eigenvalues, respectively. Because $\Lambda_{c}=M\Lambda_{\Phi }$, $V_{\Phi }=X_{\Phi c}$**A** [20], the pseudo-inverse of the estimated background covariance matrix can be expressed as:

(8) $${\hat{K}}_{b\Phi }^{\#}=V_{\Phi }\Lambda_{\Phi }^{-1}V_{\Phi }^{T}=MX_{\Phi c}K_{c}^{-1}X_{\Phi c}^{T}$$

where M is a constant, and it can be ignored. Inserting Equation (8) into Equation (5) it can be rewritten as:

(9) $$\begin{array}{l} \mathrm{KRX}(\Phi (r))=(\Phi (r)-{\hat{\mu }}_{b\Phi }{)}^{T}{\hat{K}}_{b\Phi }^{-1}(\Phi (r)-{\hat{\mu }}_{b\Phi })\\ \;=(\Phi (r)-{\hat{\mu }}_{b\Phi }{)}^{T}X_{\Phi c}K_{c}^{-1}X_{\Phi c}^{T}(\Phi (r)-{\hat{\mu }}_{b\Phi }) \end{array}$$

Due to the high dimensionality of the feature space and the nonlinear mapping function Φ are unknown, it is difficult to compute directly in the feature space. Nevertheless, kernel-based learning method uses a kernel trick that the dot products in the feature space can be replaced by a kernel function [21], which is represented as:

(10) $$k(x_{i},x_{j})=<\Phi (x_{i}),\Phi (x_{j})>=\Phi (x_{i})\cdot \Phi (x_{j})$$

Through kernel trick and derivation, then:

(11) $$\begin{array}{l} \Phi {\left(r\right)}^{T}X_{\Phi c}=\Phi {\left(r\right)}^{T}([\Phi (x_{1}),\Phi (x_{2}),\cdot \cdot \cdot ,\Phi (x_{M})]-{\hat{\mu }}_{b\Phi })\\ \;=k(r,X_{b})-\frac{1}{M}\sum_{i=1}^{M}k(r,x_{i})1_{1\times M}\\ \;\equiv k_{r}^{T} \end{array}$$

(12) $$\begin{array}{l} {\hat{\mu }}_{b\Phi }^{T}X_{\Phi c}={\hat{\mu }}_{b\Phi }^{T}([\Phi (x_{1}),\Phi (x_{2}),\cdot \cdot \cdot ,\Phi (x_{M})]-{\hat{\mu }}_{b\Phi })\\ \;=[\frac{1}{M}\sum_{i=1}^{M}{k(x}_{i},X_{b})]-[\frac{1}{M^{2}}\sum_{i=1}^{M}\sum_{j=1}^{M}k(x_{i},x_{j})]1_{1\times M}\\ \;\equiv k_{\hat{\mu }}^{T} \end{array}$$

where **1**_1__×M_ denotes *M* dimensional row vector whose elements are all 1. Due to the centralization of the feature space cannot be obtained, it calculates **K***_c_* via **K**b = Φ(**X***_b_*)^T^Φ(**X***_b_*):

(13) $$K_{c}=K_{b}-K_{b}I_{M}-I_{M}K_{b}+I_{M}K_{b}I_{M}$$

where **I***_M_* is an M-dimensional square matrix of all elements as 1/M. Finally, the KRX algorithm can be simplified as:

(14) $$\mathrm{KRX}\left(\Phi \left(r\right)\right)={\left(k_{r}^{T}-k_{\hat{\mu }}^{T}\right)}^{T}K_{c}^{-1}\left(k_{r}^{T}-k_{\hat{\mu }}^{T}\right)$$

### 2.2. WSSKRX Algorithm and Parallel Implementation on GPUs

The original KRX algorithm makes full use of the nonlinear information between bands by kernel mapping. Unfortunately, when the anomaly information is mixed into the background data, the kernel mapping cannot represent the ideal distribution of the background data, and it affects the detection performance. Furthermore, one limitation of KRX algorithm is that it has highly computational complexity because of massive nonlinear kernel function and inverse of covariance matrices. Accordingly, the novel WSSKRX detector is proposed in this paper, and a parallel architecture on GPUs is designed according to the characteristics of WSSKRX.

#### 2.2.1. The Local Information Reconstruction

**Figure 2:**
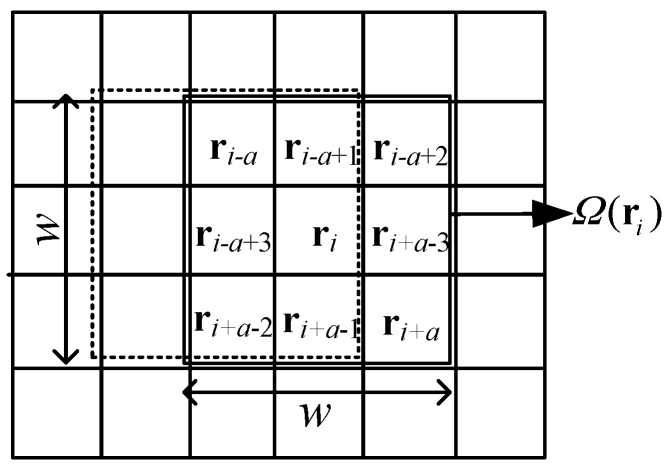
The local sliding window *Ω*(**r***_i_*).

For any pixel **r***_i_* = (*r_i_*_1_,*r_i_*_2_,…,*r_iL_*)^T^ where *L* is the total number of spectral bands, its neighborhood information is spectral correlative and spatial correlative. Assume that *Ω*(**r***_i_*) is a local sliding window with **r***_i_* being the centered pixel and the size of window being *w*^2^ = *w* × *w* where *w* is an odd number and positive integer (as shown in Figure 2). The representation form of *Ω*(**r***_i_*) is given by:

(15) $$\Omega (r_{i})=\{r_{p}|p\in [i-a,i+a]\}$$

where **r***_p_* is a pixel within the neighborhood window, and *a* = (*w*^2^ − 1)/2 is a constant. In order to better capture the spatial information, the centered pixel is reconstructed according to the weighted spatial-spectral information in this paper. The reconstructed pixel ${\hat{r}}_{i}$ is specified by:

(16) $${\hat{r}}_{i}=\frac{\sum_{r_{p}\in \Omega (r_{i})}\omega_{p}r_{p}}{\sum_{r_{p}\in \Omega (r_{i})}\omega_{p}}$$

where $\omega_{p}=\exp(-t||r_{i}-r_{p}|{|}_{2}^{2})$ is the weight of any pixel **r***_p_* to center pixel **r***_i_* in the spatial neighborhood $\Omega (r_{i})$, $||•|{|}_{2}$ denotes two norm operation. t > 0 is a spectral factor, indicating the degree of interaction effects between different pixels in the same neighboring space. Accordingly, we can get the kernel function between the reconstructed pixels as:

(17) $$\begin{array}{l} k_{SS}({\hat{r}}_{i},{\hat{r}}_{j})=<\Phi ({\hat{r}}_{i}),\Phi ({\hat{r}}_{j})>\\ \;=\langle \frac{\sum_{r_{p}\in \Omega (r_{i})}\omega_{p}r_{p}}{\sum_{r_{p}\in \Omega (r_{i})}\omega_{p}},\frac{\sum_{r_{q}\in \Omega (r_{j})}\omega_{q}r_{q}}{\sum_{r_{q}\in \Omega (r_{j})}\omega_{q}}\rangle \\ \;=\frac{\sum_{r_{p}\in \Omega (r_{i})}\sum_{r_{q}\in \Omega (r_{j})}\omega_{p}\omega_{q}k(r_{p},r_{q})}{\sum_{r_{p}\in \Omega (r_{i})}\omega_{p}\sum_{r_{q}\in \Omega (r_{j})}\omega_{q}} \end{array}$$

**Figure 3:**
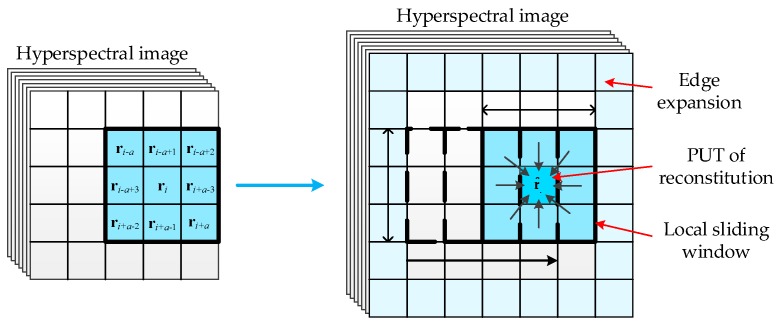
Illustration of information reconstitution.

The weight *ω_p_* is greater when the spectral curve of two pixels are closer and *ω_p_* is determined by the spectral factor *t*. Furthermore, a pixel will be incorporated into the center pixel with a smaller weight when it is distinct greatly from the center pixel in the neighborhood space. For example, if the PUT is the background pixel and anomalies exist in the background sample data, the reconstructed PUT will be closer to the background features and weaken the influence of abnormal information on background data. Therefore, the local PUT reconstruction can effectively avoid performance degradation duo to background data are contaminated by anomalous information. It should be noted that the reconstruction and detection of the edge pixels need to rely on the edge expansion of HSI. The sliding window is to achieve the reconstruction task of each pixel as shown in Figure 3.

Window size *w* and spectral factor *t* are two important parameters of information reconstruction. Generally, the selection of the parameter *w* and *t* depend on the image itself. When the object distribution is more concentrated, the spatial relationship between the pixels is more obvious, and the spectral difference is smaller. So we should choose larger *w* and smaller *t*. On the contrary, when the distribution is more dispersed, we need to choose a smaller *w* to represent weaker spatial relationship and larger *t* to weaken the impact of abnormal information.

#### 2.2.2. Weighted Spatial-Spectral Kernel RX Method

The detection performance of kernel-based method depends on the form of kernel function and the selection of kernel parameter. The effective use of spatial-spectral information in kernel-based anomaly detection is helpful to optimize the kernel mapping in high-dimensional feature space, thereby improving the performance of anomaly detection. We redefine the feature vector of the sample point ${\tilde{r}}_{i}$ which includes spatial information ${\hat{r}}_{i}$ and spectral information $r_{i}$. When the spatial and the spectral vector are constructed, the kernel function can be obtained by satisfying the kernel function of the Mercer condition [22], which is specified by:

(18) $$\{\begin{array}{c} k({\tilde{r}}_{i},{\tilde{r}}_{j})=\mu k_{1}({\hat{r}}_{i},{\hat{r}}_{j})+(1-\mu )k_{2}(r_{i},r_{j})\\ s.t.\;0\leq \mu \leq 1 \end{array}$$

where *μ* is a weighting factor. Gaussian radial basis function (RBF) kernel has a translation invariance and better capability of local information retrieval in the feature space. Therefore, in this paper, we use RBF kernel as the base kernel mapping function:

(19) $$k(x,y)=\exp(-||x-y|{|}^{2}/c)$$

where *c* is a positive constant which represents the width of the RBF kernel. According to Equations (17) and (18), $\Phi {\left(r\right)}^{T}X_{\Phi c}$ in Equation (11) can be re-expressed as:

(20) $$\begin{array}{l} \Phi {\left(\tilde{r}\right)}^{T}X_{\Phi c}=\Phi {\left(\tilde{r}\right)}^{T}([\Phi (x_{1}),\Phi (x_{2}),\cdot \cdot \cdot ,\Phi (x_{M})]-{\hat{\mu }}_{b\Phi })\\ \;=k(\tilde{r},X_{b})-\frac{1}{M}\sum_{i=1}^{M}k(\tilde{r},x_{i})I_{1\times M}\\ \;=\mu k(\hat{r},X_{b})+(1-\mu )k(r,X_{b})-\frac{1}{M}\sum_{i=1}^{M}[\mu k(\hat{r},x_{i})+(1-\mu )k(r,x_{i})]I_{1\times M}\\ \;=\mu \left[k(\hat{r},X_{b})-\frac{1}{M}\sum_{i=1}^{M}k(\hat{r},x_{i})I_{1\times M}\right]+(1-\mu )\left[k(r,X_{b})-\frac{1}{M}\sum_{i=1}^{M}k(r,x_{i})I_{1\times M}\right]\\ \;=\mu \Phi {\left(\hat{r}\right)}^{T}X_{\Phi c}+(1-\mu )\Phi {\left(r\right)}^{T}X_{\Phi c}\\ \;\equiv {\tilde{k}}_{r}^{T} \end{array}$$

where 0 ≤ *μ* ≤ 1, $\hat{r}$ is the reconstructed sample pixel of PUT, and **r** is the spectral information of PUT. Therefore, we can re-write the expression of KRX as:

(21) $$\mathrm{WSSKRX}\left(\Phi \left(\tilde{r}\right)\right)={\left({\tilde{k}}_{r}^{T}-k_{\hat{\mu }}^{T}\right)}^{T}K_{c}^{-1}\left({\tilde{k}}_{r}^{T}-k_{\hat{\mu }}^{T}\right)$$

**Figure 4:**
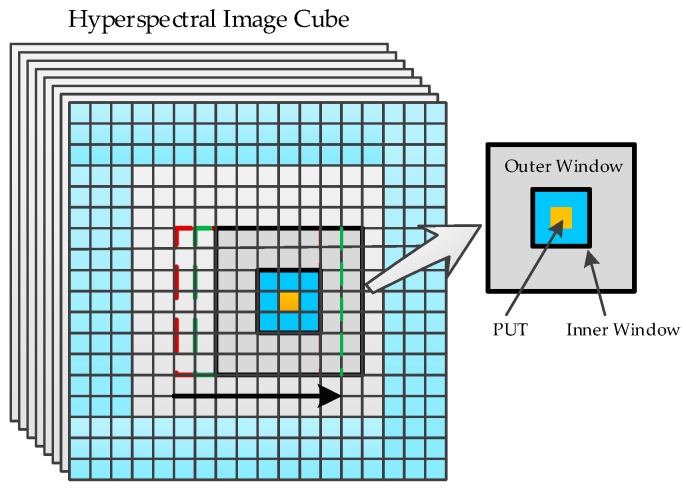
Dual concentric windows model for WSSKRX anomaly detection.

As illustrated in Figure 4, the WSSKRX algorithm utilizes a local dual concentric windows model to detect anomalies. The inner window is used to avoid the potential target information falling into the background. And PUT reconstruction, the local kernel matrix and background covariance matrix are calculated from the pixel vectors in the outer window.

#### 2.2.3. Parallel Implementation of the WSSKRX Algorithm on GPUs

A GPU is actually an array of streaming multiprocessors (SMs) in which each multiprocessor is featured by a single instruction multiple data (SIMD) architecture in each clock cycle. Each processor executes the same instruction but operates on multiple data streams. Significantly, there are multiple streaming processors (SPs) in each SM, which could be considered as many CPU cores. CPUs often run single-thread programs, and they only calculate a single data point per core, per iteration, while GPUs run in parallel by default. Thus, instead of calculating a single data point per SM, GPUs calculate 32 per SM. This gives a 32 times advantage in terms of data throughput.

**Figure 5:**
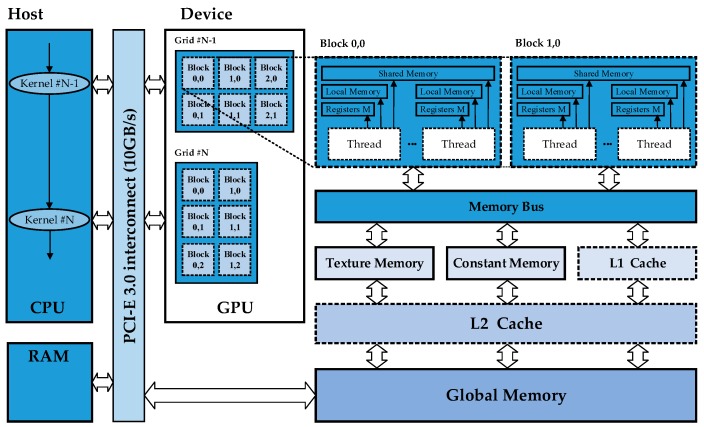
The architecture and operation of NVIDIA GPU, and the data transfer between CPU and GPU.

In Figure 5, “Host” represents the computer and “Device” represents GPUs. In device, there are different levels of memory. Thread private data will be assigned to the so-called local memory, when an excess of registers are used or the registers are depleted. In the computation, we would rather take full advantage of shared memory, which can be shared to threads in the same block to write/read quickly. The Global memory which could provide a wide memory bandwidth is supplied via Graphic Double Data Rate (GDDR) on the graphics card. It is a high-performance version of Double Data Rate (DDR) memory.

CUDA takes a simple model of data parallelism and incorporates it into a programming model without the need for graphics primitives. Being the easiest language to develop, CUDA has a huge lead in terms of maturity. CUDA was launched by NVIDIA as a kind of general computing architecture, which can enable GPU to work out many complicated calculations. In the process of a calculation, CUDA uses a grid of blocks. This can be thought as a queue (or a grid) of processes (blocks) with no inter-process communication. Each block has many threads which operate cooperatively in batches called warps.

In this paper, the parallel version of the WSSKRX on GPU for anomaly detection is given by following steps (Algorithm 1). It is noted that the underlined parts are parallel computation executed on GPU.

**Algorithm 1.** CUDA Pseudo-Code of WSSKRX1: **INPUTS**: **D**, **N**, **L** ,$w_{out}^{2}$, $w_{in}^{2}$, t, c and μ//**D** denotes a hyperspectral image with **N** pixel, **L** denotes the number of spectral bands, $w_{out}^{2}$ and $w_{in}^{2}$ denote the size of outer and inner window, respectively, t denotes spectral factor, c denotes the width of the RBF kernel, and μ denotes weighting factor.2: cudaMemcpy ();//copy the initial hyperspectral data from host to device (data communication)3: the edge expansion of **D**;//the size of expansion is determined by the $w_{out}^{2}$4: $r_{i}={\left(r_{i1},r_{i2},\cdot \cdot \cdot ,r_{iL}\right)}^{T},r_{i}\in D$;//$r_{i}$ denotes $i^{th}$ data sample vector5: **For (N)**6: Calculate ${\hat{r}}_{i}={\left({\hat{r}}_{i1},{\hat{r}}_{i2},\cdot \cdot \cdot ,{\hat{r}}_{iL}\right)}^{T}$ in $w_{out}^{2}$;//${\hat{r}}_{i}$ denotes reconstructed information of sample vector7: cudaThreadSynchronize ();//the function of thread synchronization8: Calculate ${\tilde{k}}_{r}^{T}$ and $k_{\hat{\mu }}^{T}$;//${\tilde{k}}_{r}^{T}$ and $k_{\hat{\mu }}^{T}$ denoted the kernel operations in the feature space9: cudaThreadSynchronize ();//the function of thread synchronization10: Calucalate $K_{c}=K_{b}-K_{b}*I_{M}-I_{M}*K_{b}+I_{M}*K_{b}*I_{M}$;//$K_{c}$ denoted Gram matrix, $K_{b}=\Phi {\left(X_{b}\right)}^{T}*\Phi (X_{b})$11: $K_{c}^{-1}=\mathrm{inv}(K_{c})$;12: $\mathrm{result}={\left({\tilde{k}}_{r}^{T}-k_{\hat{\mu }}^{T}\right)}^{T}*K_{c}^{-1}*\left({\tilde{k}}_{r}^{T}-k_{\hat{\mu }}^{T}\right)$;13: cudaMemcpy();//copy the hyperspectral data from device to host (data communication)14: **End for**15: **OUTPUT:** result

**Figure 6:**
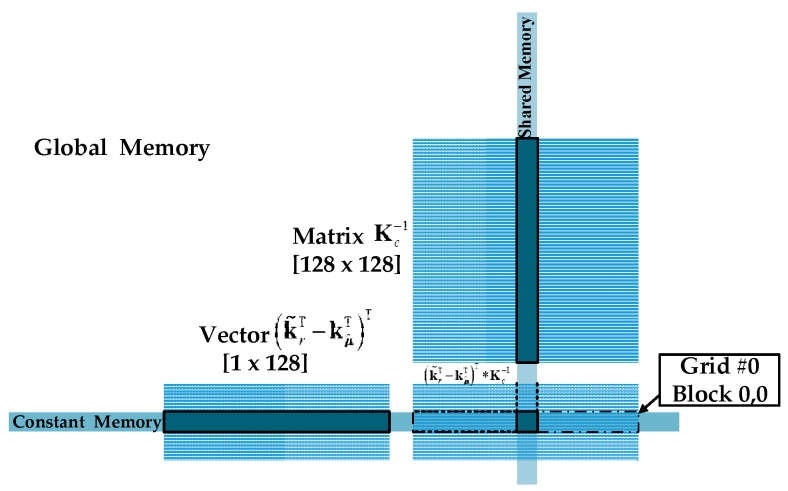
Illustration of thread for computing one element of result in GPU.

**Figure 7:**
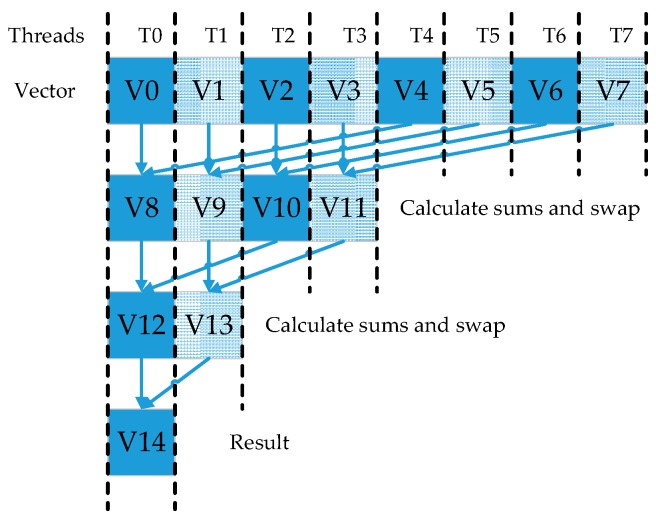
Reduction process in CUDA.

We take one or more streams as inputs and produce one or more streams as outputs by a multiprocessor. On the host, the CPU executes the kernel in CUDA, then the grid dimension is determined on the device side according to the size of matrices and the number of stream processors. Through the design of the CUDA kernel, a certain parallel task can be accomplished. Each thread will have a clear positioning by the rational allocation of thread resources, so as to achieve uniform scheduling of threads. In this implementation, we design multiple CUDA kernels, which can complete different function respectively. The following mainly introduce the four categories of CUDA kernels:

(1) In order to facilitate the detection of edge information, the original data need to be expanded. Thus, the first CUDA kernel (step 3) is designed to copy the original edge information to new extended boundaries. Row = blockIdx.x * blockDim.x + threadIdx.x and Col = blockIDx.y * blockDim.y + threaddx.y have been used to achieve the index of thread and useful threads have been systematically scheduled. Thereby realizing the parallel process of information expansion.
(2) The second CUDA kernel (step 6) fetches the PUTs from dual concentric windows and achieves the process of information reconstruction. The kernel launches as many blocks as the number of pixels for the original data presented in step 2, where each thread in one block computes an element of ${\hat{r}}_{i}={\left({\hat{r}}_{i1},{\hat{r}}_{i2},\cdot \cdot \cdot ,{\hat{r}}_{iL}\right)}^{T}$ and then stores their reconstructed information into the global memory.
(3) The kernel function takes a certain amount of computation in the algorithm and then the third kind of CUDA kernel is designed for each kernel operation in global memory. All the computational tasks are allocated to multiple threads for independent parallel implementation (steps 8–11). The CUDA kernel uses the sub-block of matrix and multi-warps independent operation to reduce memory latency and processing time. Since the calculation of the centralized Gram matrix $K_{c}$ is related to $k_{\hat{\mu }}^{T}$, a barrier should be created before calculation of $K_{c}$ to ensure correctness of the calculation. Through a series of parallel optimization design, the computational complexity of kernel function and matrix inversion are effectively reduced.
(4) And the last CUDA kernel (step 12) computes the results and completes target detection. To minimize the number of the global memory accesses and reduce access time, the result vector of ${\left(k_{r}^{T}-k_{\mu }^{T}\right)}^{T}$ and matrix $K_{c}^{-1}$ are partitioned into sub-blocks and transferred to the shared memory. Each block uses a total of 48 KB of shared memory. Figure 6 illustrates this procedure, where each thread is responsible for computing each element of ${\left(k_{r}^{T}-k_{\mu }^{T}\right)}^{T}*K_{c}^{-1}$. For every matrix multiplication, each thread in one warp is responsible for one calculation, in which a row of the matrix is multiplied by a column. For two vector multiplication, every corresponding element of them are multiplied and added in turn. It takes plenty of time and threads. One common approach to solve this problem is parallel reduction. It works by using half number of threads of the elements in the dataset. Every thread calculates the sum of products. The resultant element is forwarded to the next round. In addition, the number of threads is then reduced by half and the process repeats until there is just a single element remaining (in Figure 7). A better approach is to drop whole warps by selecting the element from the other half of the dataset.

## 3. Description of Hyperspectral Datasets

### 3.1. Synthetic Dataset

**Figure 8:**
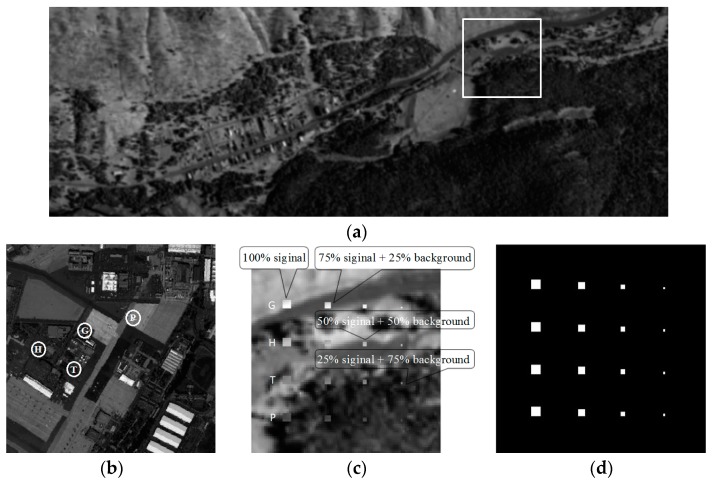
(**a**) HyMap hyperspectral image; (**b**) SanDiego Airport image; (**c**) synthetic image; (**d**) ground truth of synthetic image.

In Figure 8a, The HyMap image was acquired by HyMap hyperspectral remote sensor in the Cook City, MT, USA. There are 126 bands ranging from 0.4 to 2.5 μm with a size of 280 × 800 pixels. The background of synthetic dataset is set as the real ground which cut out the size of 90 × 90 pixels from the HyMap image (white box in Figure 8a). And the targets of synthetic dataset are designed by an airborne hyperspectral image collected by the AVIRIS imaging sensor from San Diego airport. The four targeted spectral signatures (G, H, T, P), marked by circles in Figure 8b, are used to form the synthetic targets. The four spectral signatures are used to simulate 16 targets shown in Figure 8c with 4 targets in each row simulated by same spectral signature. The sizes of panels from left column to right column are 4 × 4, 3 × 3, 2 × 2, 1 × 1, respectively. The panels in the first column are truncated from the original image, and others in 2–4 column for each row are superposed by a different proportion of background interference. And the ground truth of synthetic dataset is given by Figure 8d.

### 3.2. SanDiego Airport Dataset

The San Diego Airport dataset, collected by the AVIRIS hyperspectral spectrometer, covers the area of San Diego airport. The original data contains 400 × 400 pixels with a 3.5 m space resolution and 224 bands with a 10 nm spectral resolution (in Figure 8b).

**Figure 9:**
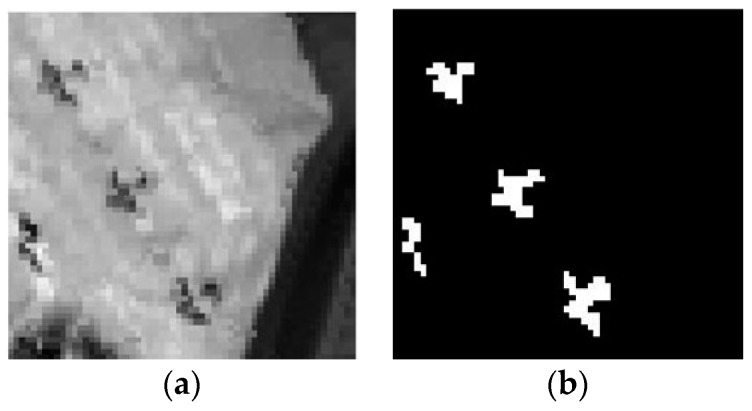
(**a**) The subarea of airport image; (**b**) ground truth of the airport image scene.

In this study, one subarea which contains 60 × 60 pixels and 126 bands is selected for experiment. In Figure 9a,b show the subarea of airport image and the ground truth of image scene, respectively.

### 3.3. SpecTIR Dataset

**Figure 10:**
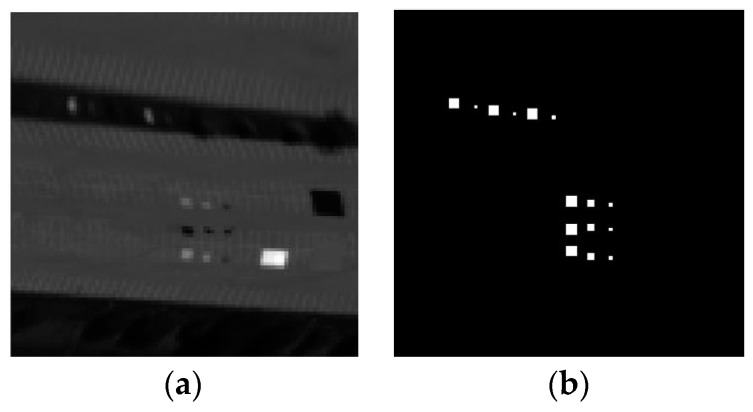
(**a**) The subarea of SpecTIR image; (**b**) ground truth of the SpecTIR image scene.

The dataset were collected through the SpecTIR Hyperspectral Airborne Rochester Experiment (SHARE) by the ProSpecTIR-VS2 sensor (SpecTIR, LLC, Rochester, New York, NY, USA). It contains 3127 × 320 pixels with an 1 m space resolution, and 360 bands ranging from 390 nm to 2450 nm with a 5 nm spectral resolution. In this experiment, a subset with a size of 100 × 100 pixels and 360 bands is segmented from the original data. There are some square fabrics placed as anomaly targets in the subarea of image (Figure 10a), and the ground truth map is shown in Figure 10b.

## 4. Experimental Results and Analysis

In this section, to investigate the anomaly detection accuracy and parallel performance, experiments are conducted one synthetic dataset and two real datasets. All the experiments are performed on a 2.0 GHz Intel Xeon E7-4820, running on a 64-bits operating system with 96 GB of RAM memory. The parallel version is implemented in CUDA C programing language for GPU cards of NVIDIA Tesla K40m. WSSKRX algorithm is compared with Global RX (GRX), Local RX (LRX), Local KRX (LKRX) and Hybrid Kernel RX (HKRX) [23] to evaluate the detection accuracy. HKRX algorithm extract the global and local feature information effectively via adding a modified spectral angle kernel to Gaussian kernel function to improve the detection performance. Other competitors have been introduced in Section 2.

### 4.1. Effects from the Parameter on WSSKRX

For the parameters of the algorithm, by virtue of 4-fold cross-validation, the RBF kenrel parameter *c* in WSSKRX on synthetic dataset, San Diego Airport dataset and SpecTIR dataset is 5, 2 and 2, respectively. The spectral factor *t* is set to 2, 2 and 10, and the weighting factor *μ* of WSSKRX is set to be 0.6, 0.5 and 0.4 on synthetic dataset, San Diego Airport dataset and SpecTIR dataset, respectively. The window size (*w_in_*,*w_out_*) is set to (3,11), (5,11) and (3,11) on three datasets, respectively.

**Figure 11:**
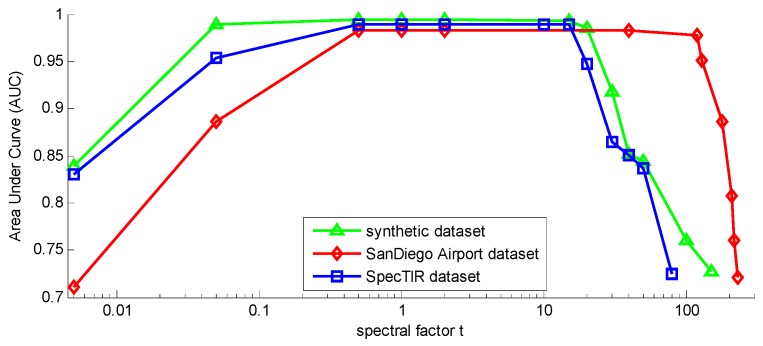
The AUC of WSSKRX with the changing spectral factor *t* on the San Diego Airport dataset, SpecTIR dataset and synthetic dataset.

Figure 11 shows the different AUC of WSSKRX with changing spectral factor *t* on three hyperspectral images. It is clear that if spectral factor *t* is less than 1 or larger than 120, the AUC descends immediately for the San Diego Airport dataset, and the corresponding AUC is the highest when t=2. For SpecTIR dataset and synthetic dataset, the AUCs tend to be stable when the spectral factor is between 1 and 30, and they reach a maximum at t=10 and t=2, respectively. Therefore, it is concluded that spectral factor *t* is not sensitive in some numerical range. Generally, when the distribution of data features are more concentrated, the selected *t* should be smaller.

**Figure 12:**
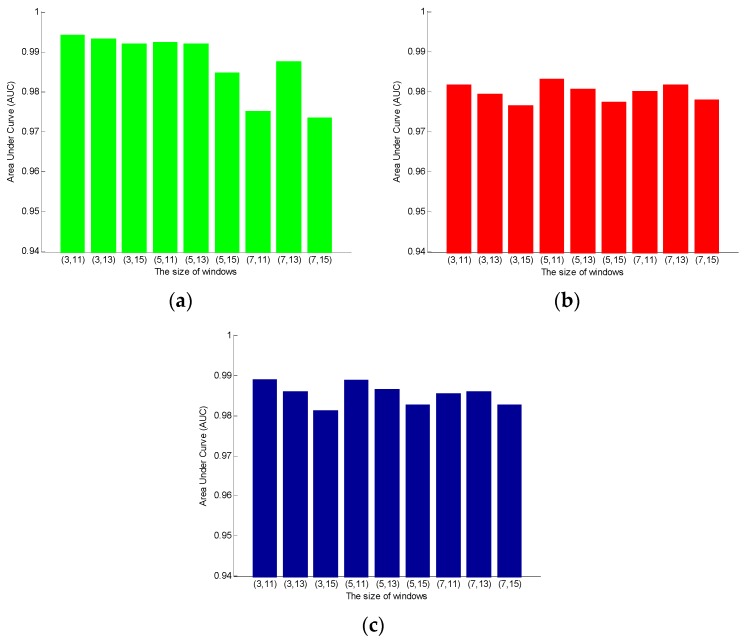
The AUC of WSSKRX with the changing window size $\left(w_{in},w_{out}\right)$. (**a**) Synthetic dataset; (**b**) San Diego Airport dataset; (**c**) SpecTIR dataset.

Figure 12 gives the AUC curves of WSSKRX using different size of dual windows on three datasets, respectively. When the size of windows on synthetic dataset, San Diego Airport dataset and SpecTIR dataset are (3, 11), (5, 11) and (3, 11) respectively, the detection performance of WSSKRX is optimal. The optimal inner window size *w_in_* used to protect anomaly information is also larger, as the anomaly target of the San Diego Airport dataset is larger than other two datasets. The window sizes in general depend on the size of target pixel in the image and local-double-window model is more suitable for small target data processing. Generally, the analysis of window sizes were considered in the range from (3, 11) to (7, 15) in the field of anomaly detection. For the RBF kernel parameter and the weighting factor, we can determine a proper parameter size by cross-validation.

### 4.2. Detection Accuracy of WSSKRX

In the experiments, for WSSKRX, all of parameters are the same as in Section 4.1. For the methods used as comparison, cross-validation is used to get optimal parameters. For LKRX and HKRX, the parameter *c* of RBF kernel is set to be 5 on synthetic dataset, 2 on two real datasets. For HKRX, the parameter *d* of spectral angle kernel is set to be 2 on synthetic and San Diego Airport dataset, 4 on SpecTIR dataset and the weighting factor *α* is set to be 0.7, 0.4 and 0.5 on three datasets, respectively. The size of dual windows in LKRX, HKRX and LRX, is the same as WSSKRX on three datasets.

**Figure 13:**
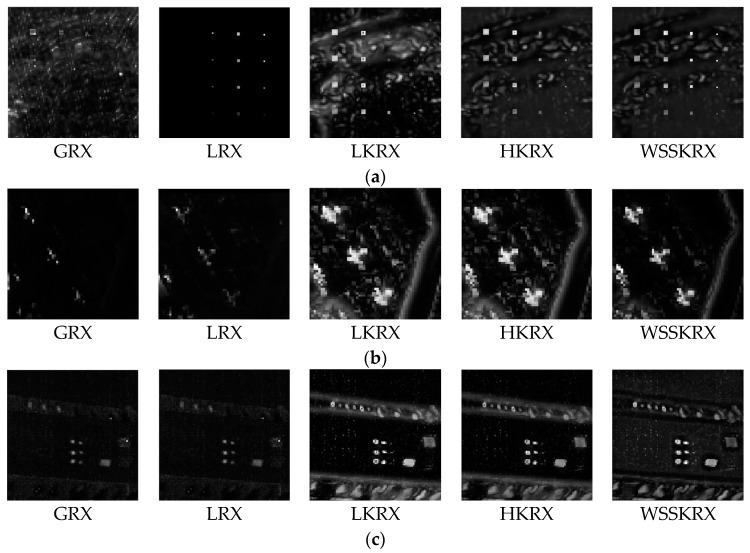
The grayscale detection results of GRX, LRX, LKRX, HKRX and WSSKRX on three datasets synthetic dataset San Diego Airport dataset SpecTIR dataset. (**a**) The grayscale of synthetic dataset; (**b**) The grayscale of San Diego Airport dataset; (**c**) The grayscale of SpecTIR dataset.

**Figure 14:**
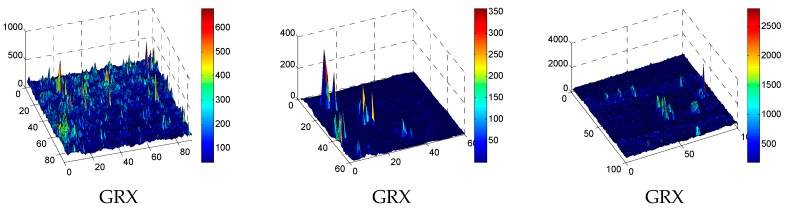

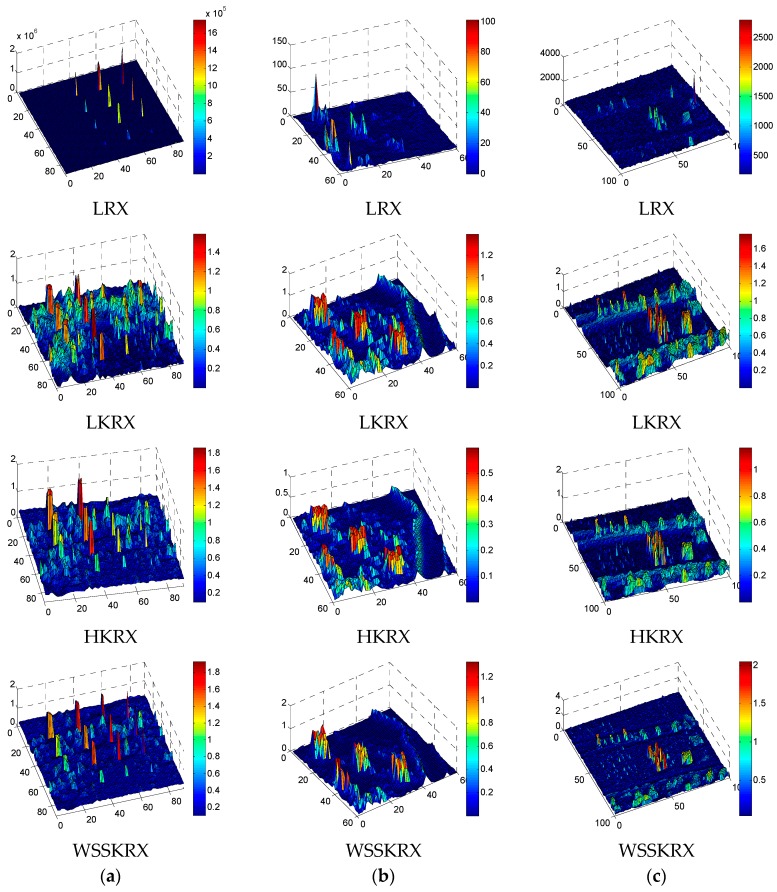
The 3D plots detection results of GRX, LRX, LKRX, HKRX and WSSKRX on three datasets. (**a**) The 3D plots of synthetic dataset; (**b**) The 3D plots of San Diego Airport dataset; (**c**) The 3D plots of SpecTIR dataset.

Figure 13 and Figure 14 show the grayscale and three-dimensional plots outputs of GRX, LRX, LKRX, HKRX and WSSKRX on three hyperspectral images, respectively. In Figure 13, LKRX, HKRX and WSSKRX can detect more abnormal pixels compared with GRX and LRX. This is because LKRX, HKRX and WSSKRX which are based on the kernel function exploit the nonlinear characteristics between spectral bands. Simultaneously, as WSSKRX algorithm effectively combines the spatial-spectral information, the detection accuracy is higher than other four detectors. In Figure 14, it can be seen that WSSKRX has better ability of suppressing noise interference compared with the LKRX and HKRX. From the observations, the separability performance of WSSKRX is better than other algorithms.

**Figure 15:**
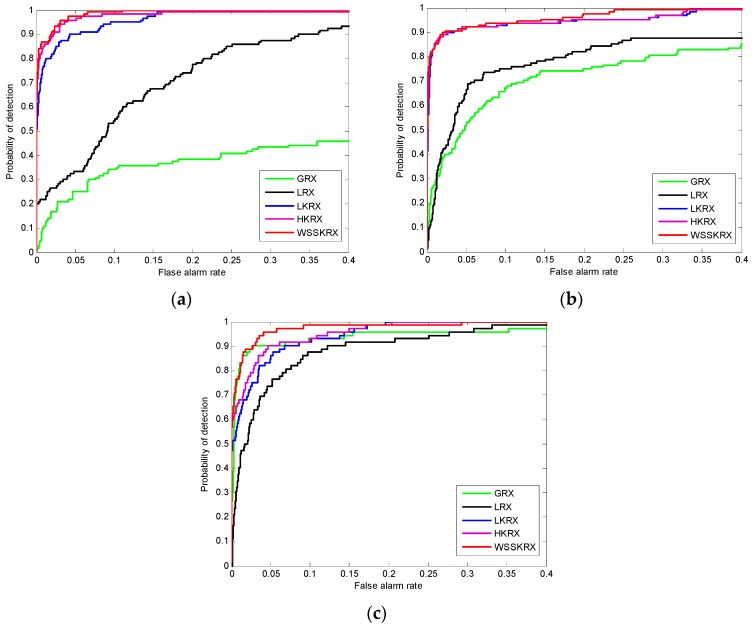
ROC curves of LRX, LKRX and WSSKRX on the two real datasets. (**a**) Synthetic dataset; (**b**) San Diego Airport dataset; (**c**) SpecTIR dataset.

In order to give a more objective evaluation, Figure 15 presents the ROC curves of GRX, LRX, LKRX, HKRX and WSSKRX on three datasets. Since the feature distribution of the synthetic dataset is more complex and the GRX and LRX are operated in low-dimensional space, they perform worse as shown in Figure 15a. The detection accuracy of HKRX is better than KRX because the global and local information is taken into account together. The WSSKRX obtains better detection performance than other detectors, because it more rationally uses the data information. In Figure 15b, GRX and LRX perform worse than other algorithms. The ROC curves of LKRX, HKRX and WSSKRX are the similar when the false alarm rate is lower than 0.06. After that, WSSKRX begins to obtain a higher detection probability than LKRX and HKRX, and it achieves 1 probability of detection with a lower false alarm rate than LKRX and HKRX. In Figure 15c, Although WSSKRX needs a slightly higher false alarm rate than LKRX and HKRX when achieving 1 probability of detection, the overall detection performance of WSSKRX is still clearly better than other algorithms. It is noted that the feature distribution of the SpecTIR dataset is more separable than other two datasets, so GRX obtains better detection performance.

### 4.3. Analysis of Parallel Performance

In this subsection, the three datasets are used to validate the improved performance of GPU version when compared with CPU version. We evaluate the parallel performance of WSSKRX in NVIDIA Tesla K40m. Table 1 shows the processing times measured for the three hyperspectral images, along with computational speedup of the implementation on GPUs and CPU for WSSKRX. The C function “clock ()” was used for timing the CPU processing, and the CUDA timer was used for GPU implementation. The time measurement was started right after the hyperspectral image loaded to CPU memory and stopped right after the results were obtained and stored in CPU memory. All the experiments are conducted 10 times and averaged to remove the computer error in Table 1.

Among the three datasets, the data amount of SpecTIR dataset is the largest and San Diego Airport dataset is the smallest. Plenty of computation in kernel maps and inverse matrices processing make the CPU version of WSSKRX time-consuming. However, it can be observed in Table 1 that a high speedup (up to 31.763) is achieved by the GPUs implementation of which processing time is much lower than the CPU version. The advantage of the implementation on GPUs could be more distinct with the increase of the data size. This is mainly due to the fact that a much larger range of data can be executed effectively.

To sum up, the experiments reported on Table 1 indicate that the GPUs can significantly improve the computational performance of considered target detection algorithms. It provides speedup on the order 13.815 for San Diego Airport dataset, on the order 25.208 for synthetic dataset, and on the order 31.763 for SpecTIR dataset. Although the proposed implementations can still be optimized, significant speedups can be obtained when the algorithms are applied on GPUs.

## 5. Discussion

In the previous section, the simulation and experiment results are analyzed. It is found that the WSSKRX algorithm effectively improves the detection accuracy. This is mainly due to the introduction of the spatial information. Reconstruction of the PUT can balance the relationship between neighborhood pixels via different weights, thereby decrease the influence of background noise. As the growth of the spatial resolution in HSI data, better use of spatial information will be the trend which is the field of anomaly detection.

The calculation principles of GPUs is parallelization of a large number of tasks to achieve and gains ideal parallel efficiency. The results demonstrated that parallel speedup increased as the increase of data size. The recent explosion in the amount and dimensionality of hyperspectral data, calls for the incorporation of parallel computing techniques to accelerate the time-consuming algorithms. Therefore, it is of great academic significance and valuable to do research on GPUs. Generally, the algorithm with spatial has high computational complexity, and by virtue of GPUs, it will obtain a good detection performance.

## 6. Conclusions and Future Research Lines

In this paper, a new weighted spatial-spectral information kernel RX algorithm (WSSKRX) and its parallel implementation on GPUs is presented. The proposed algorithm effectively reduces the interference of the background noise in the detection by utilizing spatial-spectral information rationally, which makes the detection performance better. Taking advantage of the designed parallel systems, the GPU versions of WSSKRX algorithm provide significant speedup when compared with CPU versions. The speedup is more distinct with the increase of the amount of data. Experimental results was oriented towards analyzing the anomaly target detection accuracy and parallel performance with synthetic and real hyperspectral images. In future work we will continue to modify the algorithm to improve its accuracy and explore additional strategies for better computing performance. Other architectures, such as Digital Signal Processors (DSPs) and Field-Programmable Gate Array (FPGA), will be also applied due to their capacity of onboard high performance hyperspectral data processing systems.

## Figures and Tables

**Table 1:** Processing Time (In Seconds) for CPU and GPU Implementation of WSSKRX.

	San Diego Airport Dataset	Synthetic Dataset	SpecTIR Dataset
CPU/s	154.097	411.388	655.076
GPU/s	11.154	16.320	20.624
Speedup	13.815	25.208	31.763
